# Serum bone markers in ROD patients across the spectrum of decreases in GFR: Activin A increases before all other markers 

**DOI:** 10.5414/CN109650

**Published:** 2019-03-13

**Authors:** Florence Lima, Hanna Mawad, Amr A. El-Husseini, Daniel L. Davenport, Hartmut H. Malluche

**Affiliations:** 1 Division of Nephrology, Bone and Mineral Metabolism, and; 2 Department of Surgery, University of Kentucky, Lexington, KY, USA

**Keywords:** activin A, CKD-MBD, bone turnover, renal osteodystrophy

## Abstract

Introduction: Renal osteodystrophy (ROD) develops early in chronic kidney disease (CKD) and progresses with loss of kidney function. While intact parathyroid hormone (PTH), 1,25-dihydroxyvitamin D3 (1,25D), and fibroblast growth factor-23 (FGF-23) levels are usually considered the primary abnormalities in ROD development, the role of serum activin A elevations in CKD and its relationships to ROD have not been explored. The aims of this study were to evaluate serum activin A at different CKD stages, and to establish the relationships between activin A, bone biomarkers, and bone histomorphometric parameters. Materials and methods: 104 patients with CKD stages 2 – 5D underwent bone biopsies. We measured in the serum activin A, BSAP, DKK1, FGF-23, α-Klotho, intact PTH, sclerostin, TRAP-5b, and 1,25D. Biochemical results were compared across CKD stages and with 19 age-matched controls with normal kidney function. Results: Median activin A levels were increased in all stages of CKD compared to controls from 544 pg/mL in CKD 2 (431 – 628) to 1,135 pg/mL in CKD 5D (816 – 1,456), compared to 369 pg/mL in controls (316 – 453, p < 0.01). The increase of activin A in CKD 2 (p = 0.016) occurred before changes in the other measured biomarkers. Activin A correlated with intact PTH and FGF-23 (r = 0.65 and 0.61; p < 0.01) and with histomorphometric parameters of bone turnover (BFR/BS, Acf, ObS/BS and OcS/BS; r = 0.47 – 0.52; p < 0.01). These correlations were comparable to those found with intact PTH and FGF-23. Conclusion: Serum activin A levels increase starting at CKD 2 before elevations in intact PTH and FGF-23. Activin A correlates with bone turnover similar to intact PTH and FGF-23. These findings suggest a role for activin A in early development of ROD.

## Introduction 

In the US, there are ~ 30 million patients with chronic kidney disease (CKD). The majority of these patients has chronic kidney disease mineral and bone disorder (CKD-MBD) which represents a pervasive health problem [1]. CKD-MBD presents with dysregulated mineral metabolism, increased risk for bone fracture, cardiovascular calcification, left ventricular hypertrophy, and increased mortality [2, 3, 4, 5, 6]. Renal osteodystrophy (ROD) represents the bone manifestation of CKD-MBD; it starts in patients as early as CKD stage 2 and progresses with further loss of kidney function [7, 8]. Virtually all patients requiring replacement of kidney function by dialysis have evidence of renal osteodystrophy. Progressive loss of kidney function is associated with an increase in intact parathyroid hormone (PTH), serum phosphorus, fibroblast growth factor-23 (FGF-23), and a decrease in 1,25-dihydroxyvitamin D3 (1,25D). These abnormalities are considered to be the main pathologic factors for renal osteodystrophy [7, 9, 10], but they are not sufficient to explain the bone changes that may occur as early as stage 2 [7, 11]. Identification of novel factors, especially in the early stages of CKD, is important for a more complete understanding of the pathogenesis of ROD. 

Activin A, a multifunctional cytokine [12], has recently been studied in experimental animals with reduced kidney function [13, 14]. It is the most abundant of the transforming growth factor-beta (TGF-β) family of protein found in bone matrix [15]. Moreover, its expression has been shown to be coupled with bone resorption [16] and inhibition of activin signaling results in stimulation of bone growth [13]. Activin receptor type II A inhibition by the ligand trap RAP011 was shown to inhibit osteoclast formation in vitro and bone remodeling in CKD diabetic mice [13]. These data point to a potentially important role for activin in bone turnover. Bone turnover abnormalities are an integral part of pathologic features of renal osteodystrophy, and therefore it appears important to study blood levels of activin A in patients across the spectrum of loss of glomerular filtration rate (GFR). 

The aims of this study were to: 1) evaluate serum activin A levels in patients at different stages of CKD, 2) compare activin A levels with other known biomarkers of ROD at different stages of CKD, and 3) establish the relationships between histomorphometric parameters of ROD and serum levels of activin A versus the other known ROD bone markers. 

## Material and methods 

### Patients 

This is a cross-sectional study of 104 CKD patients, stage 2 – 5D (on dialysis) who agreed to undergo bone biopsy for research purposes or workup for bone loss diagnosed by DXA. Before biopsy, for bone labeling, demeclocycline hydrochloride (150 mg b.i.d.) was administered for 2 days and tetracycline hydrochloride (250 mg b.i.d.) for 4 days, each separated from the other by a period of 10 days. Biopsies were performed 3 days after completion of the second label. All patients had blood draw at time of the bone biopsy. Inclusion criteria were age ≥ 18 years with a diagnosis of CKD stages 2 – 5D. Exclusion criteria were history of renal transplantation, history of parathyroidectomy, use of medications known to affect bone metabolism (except for calcitriol or cinacalcet), and life-threatening comorbid conditions such as malignancy, active infection, and hepatic disease. In addition, blood was drawn during the same time period from 19 individuals with normal kidney function who served as controls. These individuals were not receiving any anti-osteoporosis drugs at the time of the biopsy and during 2 years before biopsy. Informed consent was signed by all patients, and the study was reviewed and approved by the Institutional Review Board at the University of Kentucky. The study was conducted according to the Declaration of Helsinki. The patients’ medical records were reviewed to obtain demographic data, medication usage comorbidities, and data about dialysis vintage. 

### Serum biochemistry 

Activin A levels were measured using R&D Systems kits (Indianapolis, ID, USA), sclerostin and DKK1 levels using Biomedica kits (Vienna, Austria), FGF-23 using Kainos kits (Tokyo, Japan), α-Klotho using IBL kits (Fujioka-Shi, Gunma, Japan), BSAP and TRAP-5b using Quidel kits (San Diego, CA, USA). Intact PTH and 1,25D were measured using chemiluminescence analyzers (DiaSorin, Stillwater, MN, USA). Serum creatinine, calcium, and phosphorus levels were measured by automated techniques. All measurements were performed in duplicate. Estimated GFR (eGFR) was determined using the MDRD formula. 

### Mineralized bone histology and bone histomorphometry 

Bone samples were obtained by bone biopsies of the anterior iliac crest under local anesthesia and sedation. They were fixed in ethanol at room temperature, dehydrated, and embedded in methyl methacrylate as described previously [17]. Sections were stained with the modified Masson-Goldner trichrome stain [18], the aurin tricarboxylic acid stain [19], and solochrome azurine stain [20]. Unstained sections were prepared for phase-contrast and fluorescence light microscopy. Bone histomorphometry for static and dynamic parameters of bone structure, formation, and resorption was done at a magnification of 200 × using the OsteoMeasure (OsteoMetrics, Atlanta, GA, USA). All measured histomorphometric parameters are in compliance with the recommendations of the nomenclature committee of the American Society of Bone and Mineral Research [21, 22]. 

### Statistical analyses 

Results were reported as means (±SD) or medians (25^th^ – 75^th^ quartiles, IQR) when values were not normally distributed. Categorical variables were expressed as percentages. Comparisons of continuous variables were done using Kruskal-Wallis or Mann-Witney U-tests as appropriate. Correlations between activin A and other biochemical parameters, and between activin A and bone histomorphometric parameters were examined using Spearman’s rho (ρ) tests. Cutoff values for determination of low vs. non-low and high vs. non-high bone turnover were obtained by using the Youden’s J statistic. All statistical analyses were performed using SPSS version 24 (IBM Corp., Armonk, NY, USA). Group comparisons with p < 0.05 were considered statistically significant, and p < 0.005 was considered statistically significant for multiple correlations. 

## Results 

There were 104 patients, consisting of 75 females and 29 males with mean age of 59 (± 15) years. 22 patients were in CKD stage 2, 29 patients in CKD stage 3, 19 patients in stages 4 or 5, and 34 patients on maintenance hemodialysis (CKD 5D). Disease etiologies included: 18% diabetes, 11% hypertension, 4% glomerulonephritis, 2% polycystic kidney disease, 30% other etiologies, and 34% unknown. Clinical and biochemical characteristics of the patients are shown in Table 1. Biochemical parameters such as phosphorus, calcium, BSAP, and TRAP-5b were significantly higher in CKD 5D compared to other CKD groups. 

### Activin A, sclerostin, and other biochemical parameters across CKD stages 

**Figure 1.**
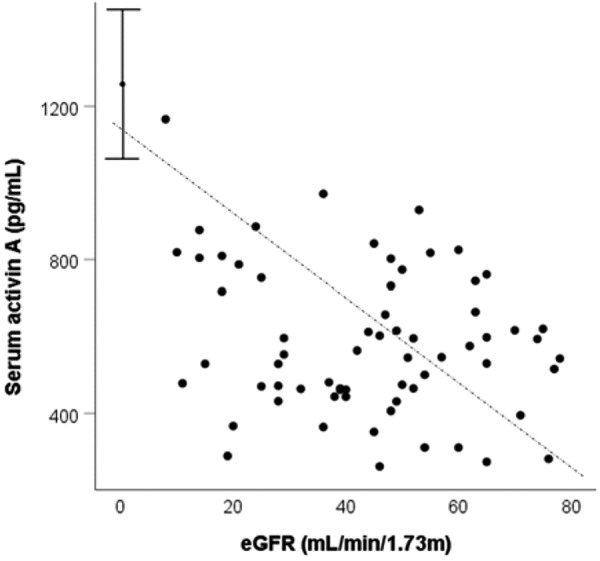
Relationships between serum levels of activin A and levels of eGFR. Results of patients with CKD 5D (n = 34) are given by mean ± 95% confidence intervals. Dashed line represents linear regression (R^2^ = 0.316, p < 0.01).

**Figure 2.**
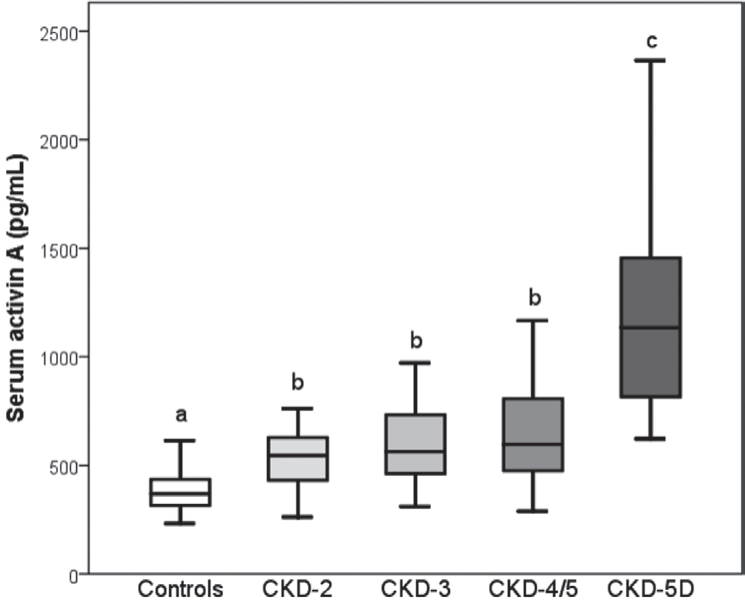
Serum levels of activin A in patients with CKD stages 2 – 5D. Results sharing the same letters are not significantly different.

Serum activin A levels increased with declining eGFR (Figure 1) (ρ = 0.580, p < 0.001). Compared to controls, median levels of activin A were significantly elevated in CKD 2 (p = 0.016) (Figure 2). There was a trend to a further increase without significant differences between CKD 2, 3, and 4/5, while in CKD 5D there was a further significant increase in serum activin A concentrations. There were no significant differences in activin A levels between diabetics and non-diabetics and patients with or without active vitamin D metabolites among the patients with stages 2 – 5 (p = 0.911 and 0.290, respectively), as well as in CKD 5D patients (p = 0.800 and 0.276, respectively). The results on changes of activin A in patients with CKD versus controls were not altered by exclusion of patients with diabetes or vitamin D treatment. 

**Figure 3.**
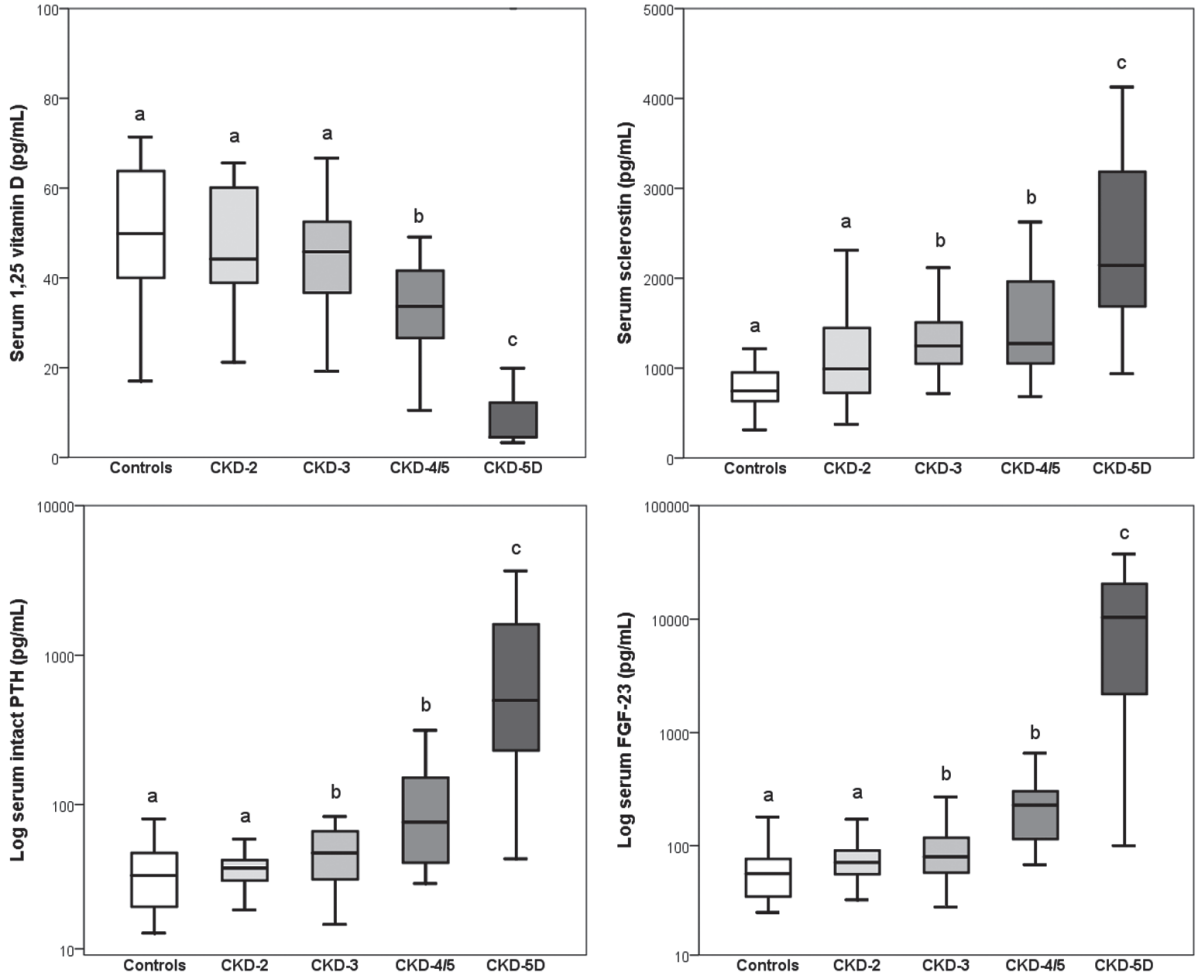
Serum levels of sclerostin, intact PTH, FGF-23, and 1,25D in patients with CKD stages 2 – 5D. Results sharing the same letters are not significantly different.

Compared to controls, median levels of sclerostin and intact PTH became elevated in CKD 3 (p = 0.018 and 0.022, respectively) (Figure 3), while median levels of 1,25D fell and FGF23 levels increased in CKD 4/5 (p = 0.008 and p < 0.001, respectively) (Figure 3). 

Across all CKD patients, serum activin A levels correlated (in descending orders) with intact PTH, FGF-23, TRAP-5b, phosphorus, 1,25D, BSAP and sclerostin (Table 2). Serum sclerostin levels also correlated in descending order with 1,25D, phosphorus, FGF-23, intact PTH, and to a lesser extent with TRAP-5b. 

### Relationships between activin A, other serum biochemical bone markers, and histomorphometric results (Table 3) 

Across all CKD stages, serum activin A levels correlated with parameters of bone formation and resorption such as activation frequency, bone formation rate, osteoblast surface, osteoclast surface, osteoid thickness, and cortical porosity. These correlations were similar to those found between intact PTH, FGF-23, and histomorphometric bone parameters. The correlations between sclerostin and the histomorphometric bone parameters were less strong and there were no significant correlations with activation frequency and osteoblast surface. DKK1 correlated with osteoid surface only while there was no correlation between histomorphometric bone parameters and α-Klotho. Intact PTH, FGF-23, 1,25D, BSAP, and TRAP-5b showed the expected relationships with histomorphometric parameters of bone formation and resorption. 

### Prediction of bone turnover by activin A and other serum biochemical bone markers 

**Figure 4.**
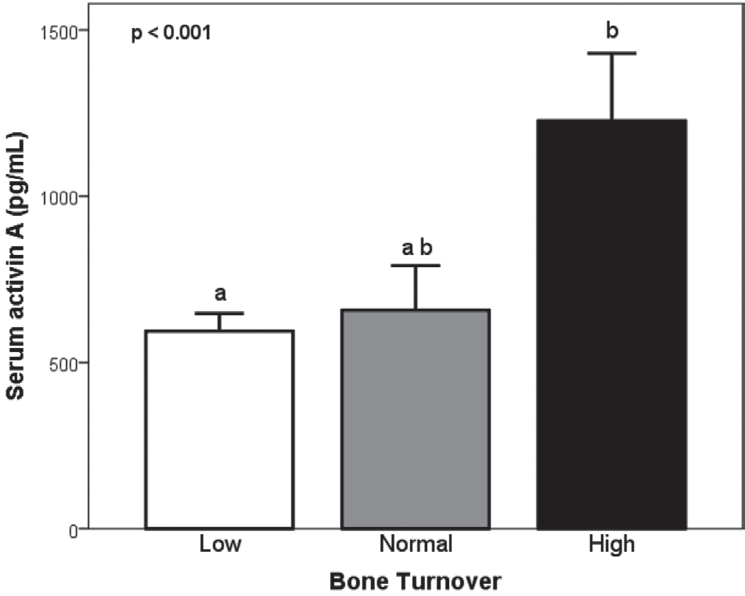
Levels of serum activin A in low, normal, and high bone turnover patients with CKD from stage 2 to 5D (group means + 95% confidence intervals). Results sharing the same letters are not significantly different.

**Figure 5.**
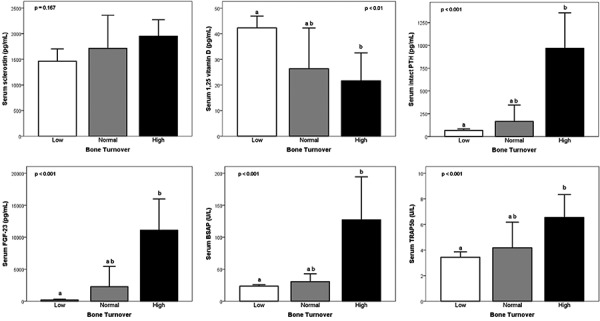
Levels of serum biochemical parameters in patients (CKD from stage 2 to 5D) with low, normal, and high bone turnover (group means + 95% confidence intervals). Results sharing the same letters are not significantly different.

Patients were classified as having low, normal, and high bone turnover based on reference values for activation frequency, bone formation rate, and numbers of osteoclasts and osteoblasts [23, 24, 25]. There were 57 subjects with low bone turnover (55%), 13 with normal (13%), and 34 with high bone turnover (33%). Levels of activin A, sclerostin, intact PTH, FGF-23, 1,25D, BSAP, and TRAP-5b separated by bone turnover are shown in Figures 4 and 5. Serum levels of activin A were significantly different between bone turnover states (ANOVA p < 0.001) (Figure 4). The other markers also varied significantly (ANOVA p’s < 0.01) (Figure 5), except for sclerostin. Activin A showed similar AUC results, specificity, and sensitivity in predicting high turnover as intact PTH, BSAP, and FGF-23 (Table 4). Vitamin 1,25D and TRAP-5b showed less sensitivity and specificity for identification of bone turnover. 

## Discussion 

The current data demonstrate the novel findings of a significant increase in activin A blood levels as early as CKD stage 2. Compared to controls, intact PTH and sclerostin increase significantly at CKD stage 3, while FGF-23 does not increase significantly and 1,25D decreases significantly at stage 4/5. The PTH findings are in agreement with several prior studies [7, 10]. Sclerostin has been recently shown to increase at CKD stage 3 [11] [26]. In agreement with our results, Gutierrez et al. [4, 9] found FGF-23 to increase in some patients at stage 3 with a significant increase at stage 4, while Isakova et al. [27] studying 3,879 participants found significant increases already at stage 3. Our results on 1,25D changes with CKD are in agreement with Levin et al. [10] who showed in a study of 1,814 CKD patients similar median levels of 1,25D by CKD stages. PTH, FGF-23, and 1,25D are commonly considered to be contributors to the pathogenesis of ROD [28, 29, 30, 31, 32], and sclerostin might play a role in the bone loss of ROD [33]. Our findings ascribe a role to activin A as the earliest documentable serum abnormality in the development of CKD-MBD; they open up a promising new avenue for research addressing the early pathogenesis of, and possible therapeutic approaches to, ROD. 

The strong associations between activin A and intact PTH, FGF-23, and 1,25D, factors involved in bone formation, resorption and mineralization, show that activin A appears to be associated with bone turnover. This is corroborated by the observation that activin A showed similar specificity as intact PTH and FGF-23 in the discrimination of high versus non-high turnover. In experimental animals with reduced kidney function [13, 14], activin A expression has been shown to be coupled with bone resorption [16] and inhibition of activin signaling results in stimulation of bone growth [13]. Activin A also has been shown to enhance osteoclast activity, and activin receptor type II A inhibition by the ligand trap, RAP011, was shown to inhibit osteoclast formation in vitro and bone remodeling in diabetic mice with CKD [13]. In mice with CKD-MBD induced by Alport syndrome, RAP011 decreased elevated osteoclast numbers and bone resorption; importantly, osteoblast numbers were not decreased, and the reduced bone formation rate per osteoblast associated with CKD-MBD was corrected [14]. Taken together, these data point to an important role for activin in bone turnover. 

Activin A showed no relationship with Klotho or DKK1 neither of which varied with CKD stages. Thus, activin A appears to be independent of α-Klotho or DKK1 in ROD. The α-Klotho results are in contrast with experimental results in animals and clinical observations in patients with acute kidney injury [34] and CKD [35]. Akimoto et al. [36, 37], using the same assay as employed in our study, found in agreement with our results no significant changes in α-Klotho and DKK1 by CKD stage. The DKK1 results are in agreement with human studies in CKD [33, 38] but in contrast with findings of elevated DKK1 in a mouse model of CKD stage 5 [39]. These discrepancies in α-Klotho and DKK1 results might be related to differences in the employed assays. 

Limitations of the study are given by description of correlations that cannot establish causality. However, in mice with CKD-MBD, activin receptor type II A inhibition by the ligand trap RAP011 prevented development of renal osteodystrophy, that is, there was correction of high bone turnover and improvement of osteoblastic function [14]. Moreover, use of the natural antagonist of activin A, inhibin, in mice resulted in an increase in bone mineral density (BMD) [40]. Taken together, these data point to an important role for activin in renal osteodystrophy. 

Further limitations of the study are related to its cross-sectional nature. Even though the total number of 104 subjects studied including bone biopsies is respectable, when broken down into CKD stages, the number of patients in each group is relatively small and does not allow analysis regarding diagnostic value of serum parameters for low versus high turnover and the role of factors such as diabetes and specific therapies. The present results provide justification for a prospective, multicenter, long-term study with bone biopsies in a larger number of patients. Currently available assays for novel markers such as α-Klotho and DKK1 are still undergoing refinements and standardizations, and could limit interpretation and comparisons of results across publications including ours. 

In conclusion, activin A is a novel player observed in ROD in addition to or independent of the known abnormalities in PTH, FGF-23, 1,25D, and sclerostin. Activin A levels increase in blood of patients with CKD starting as early as stage 2 and the relationships with bone turnover abnormalities are as strong as those found with PTH and FGF-23. The present findings open up a new avenue for research in animals and subsequently in humans addressing the early pathogenesis of ROD and potential new therapeutic approaches to this serious abnormality of CKD-MBD. 

## Funding 

This study was funded in part by the National Institutes of Health, Grant RO1 080770, UK Center for Clinical and Translational Science (CCTS), and the Kentucky Nephrology Research Trust (KNRT). 

## Conflict of interest 

The authors declare no potential conflict of interest. 

**Table 1.** Demographic, clinical, and serum biochemical parameters in patients with CKD stages 2 – 5D and controls.

	Control	CKD 2	CKD 3	CKD 4/5	CKD 5D	p-value
No. of subjects	19	22	29	19	34	
Age	55 ± 12^a,b^	65 ± 10^a^	68 ± 12^a^	60 ± 13^a,b^	49 ± 13^b^	< 0.001
Dialysis vintage, median mos. (25^th^, 75^th^)					64 (48-119)	
Patients with diabetes, n (%)	0	4 (18%)	3 (10%)	9 (47%)	11 (32%)	
Tx with active vitamin D metabolites, n (%)	2 (11%)	3 (14%)	1 (3%)	6 (32%)	21 (62%)	
Calcium containing P binders, n (%)	0	0	1 (3%)	2 (11%)	20 (59%)	
Sevelamer and lanthanum, n (%)	0	0	0	1 (5%)	12 (35%)	
Calcium (mg/dL)	9.5 ± 0.4^a^	9.6 ± 0.5 ^a^	9.6 ± 0.6^a^	9.1 ± 1.2^ab^	8.8 ± 1.0^b^	< 0.001
Phosphorus (mg/dL)	3.6 ± 0.6^a^	3.5 ± 0.8 ^a^	3.7 ± 0.4^a^	4.0 ± 0.6^ab^	5.9 ± 2.0^b^	< 0.001
Activin A (pg/mL)	369 (316 – 453)^a^	545 (431 – 628)^b^	564 (462 – 732)^b^	597 (472 – 810)^b^	1,125 (816 – 1,456)^c^	
Sclerostin (pg/mL)	744 (579 – 900)^a^	978 (697 – 1,371)^a^	1,248 (1,049 – 1,508)^b^	1,255 (986 – 1,963)^b^	2,145 (1,687 – 3,184)^c^	
PTH (pg/mL)	33 (20 – 47)^a^	37 (30 – 42)^a^	47 (31 – 66)^b^	76 (37 – 162)^b^	501 (231 – 1,612)^c^	
FGF-23 (pg/mL)	57 (37 – 76)^a^	71 (58 – 87)^a^	80 (58 – 118)^a^	229 (122 – 319)^b^	10,348 (2,021 – 23,462)^c^	
1,25D (pg/mL)	50 (40 – 64)^a^	44 (39 – 60)^a^	46 (37 – 53)^a^	34 (26 – 44)^b^	< 4.5^c^	
BSAP (U/L)	27 (21 – 32)^a^	23 (19 – 28)^a^	24 (18 – 29)^a^	23 (17 – 30)^a^	80 (36 – 134)^b^	< 0.001
TRAP5b (U/L)	3.6 (2.4 – 4.5)^a^	2.9 (2.4 – 3.8)^a^	3.1 (2.5 – 3.8)^a^	2.4 (1.8 – 4.1)^a^	5.4 (4.4 – 10.7)^b^	< 0.001
DKK1 (pmol/L)	40 (30 – 46)	32 (25 – 53)	41 (34 – 48)	23 (20 – 30)	30 (13 – 41)	0.879
α-Klotho (pg/mL)	720 (589 – 851)	837 (522 – 947)	669 (558 – 832)	691 (467 – 945)	530 (337 – 699)	0.083

Results sharing the same superscript letters are not significantly different.

**Table 2.** Correlation coefficients (ρ) between serum biochemical results in patients with CKD stages 2 – 5D.

Spearman’s ρ	Activin A	Sclerostin	Intact PTH	FGF–23	1,25D	BSAP	TRAP–5b	Phosphorus	Calcium	DKK1
Sclerostin	0.39**									
Intact PTH	0.65**	0.30**								
FGF-23	0.61**	0.33**	0.67**							
1,25D	–0.52**	–0.36**	–0.57**	–0.71**						
BSAP	0.51**	0.22	0.62**	0.40**	–0.51**					
TRAP-5b	0.52*	0.27**	0.44**	0.19	–0.38**	0.61**				
Phosphorus	0.54**	0.33**	0.45**	0.52**	–0.45**	0.28	0.29**			
Calcium	–0.18	–0.11	–0.34**	–0.23	0.41**	–0.20	–0.06	–0.26		
DKK1	–0.14	0.06	–0.27	–0.22	0.20	–0.06	0.04	–0.09	0.08	
α-Klotho	–0.07	–0.14	–0.19	–0.23	0.23	–0.06	0.01	–0.12	0.14	0.14

*p > 0.05; **p >0.01.

**Table 3.** Correlation coefficients (ρ) between serum biochemical results and bone histomorphometric parameters in patients with CKD stages 2 – 5D.

Spearman‘s ρ	Activin A	Sclerostin	Intact PTH	FGF-23	1,25D	BSAP	TRAP-5b	Phosphorus	Calcium	DKK1	α-Klotho
Bone formation rate/ Bone surface	0.51**	0.28**	0.59**	0.55**	–0.42**	0.55**	0.35**	0.43**	–0.27**	–0.22	–0.25
Activation frequency	0.47**	0.18	0.53**	0.51**	–0.38**	0.50**	0.31**	0.37**	–0.19	–0.26	–0.25
Osteoblast surface/ Bone surface	0.49**	0.16	0.58**	0.54**	–0.45**	0.39**	0.23	0.42**	–0.17	–0.24	–0.10
Osteoclast surface/ Bone surface	0.52**	0.35**	0.64**	0.55**	–0.50**	0.57**	0.43**	0.48**	–0.36**	–0.18	–0.21
Osteoid surface/ Bone surface	0.52**	0.32**	0.64**	0.54**	–0.41**	0.46**	0.30**	0.40**	–0.32**	–0.36**	–0.21
Mineralization lag time	0.20	0.13	0.25	0.12	–0.15	0.13	0.11	0.12	–0.17	–0.13	–0.05
Bone volume/ Tissue volume	0.15	0.17	0.24	0.18	–0.35**	0.31**	0.18	0.12	–0.33**	0.11	–0.08
Trabecular thickness	–0.03	0.10	0.04	0.06	–0.21	0.22	–0.01	0.02	–0.12	0.16	–0.03

**p > 0.01.

**Table 4.** Cutoff values using the maximum Youden’s J statistic and AUC of circulating biomarkers to distinguish low and high bone turnover from non-low and non-high bone turnover in patients with CKD stages 2 – 5D.

	Bone turnover							
	Low vs. non low				High vs. non high			
	Cutoffs	ROC AUC (95% CI)	Sens.	Spec.	Cutoffs	ROC AUC (95% CI)	Sens.	Spec.
Activin A (pg/mL)	677	0.80 (0.71 – 0.88)	0.79	0.72	937	0.87 (0.79 – 0.94)	0.62	0.96
Sclerostin (pg/mL)	1,670	0.64 ( 0.53 – 0.75)	0.54	0.77	1,670	0.66 (0.54 – 0.77)	0.61	0.74
PTH (pg/mL)	93	0.84 (0.76 – 0.92)	0.72	0.87	243	0.86 (0.78 – 0.95)	0.66	0.94
FGF-23 (pg/mL)	245	0.85 (0.77 – 0.92)	0.65	0.89	1,339	0.86 (0.78 – 0.94)	0.64	0.96
1,25D (pg/mL)	27	0.78 (0.67 – 0.89)	0.87	0.71	14	0.73 (0.60 – 0.87)	0.9	0.62
BSAP (U/L)	27	0.81 (0.71 – 0.90)	0.79	0.7	35	0.86 (0.77 – 0.95)	0.72	0.84
TRAP-5b (U/L)	4.3	0.66 (0.53 – 0.78)	0.56	0.8	4.3	0.68 (0.53 – 0.83)	0.65	0.76
